# Profiling the cell-specific small non-coding RNA transcriptome of the human placenta

**DOI:** 10.21203/rs.3.rs-5953518/v1

**Published:** 2025-02-12

**Authors:** Nikita Telkar, Desmond Hui, Maria S. Peñaherrera, Victor Yuan, Victor D. Martinez, Greg L. Stewart, Alexander G. Beristain, Wan L. Lam, Wendy P. Robinson

**Affiliations:** British Columbia Children’s Hospital Research Institute; British Columbia Children’s Hospital Research Institute; British Columbia Children’s Hospital Research Institute; British Columbia Children’s Hospital Research Institute; IWK Health Centre; British Columbia Cancer Research Institute; British Columbia Children’s Hospital Research Institute; British Columbia Cancer Research Institute; University of British Columbia

**Keywords:** placenta, small non-coding RNA, miRNA, trophoblast, chorionic villi

## Abstract

The human placenta is the composite of multiple cell types, each which contributes uniquely to placental function. Small non-coding RNAs (sncRNAs) are regulators of gene expression and can be cell-specific. The sncRNA transcriptome of individual placental cell types has not yet been investigated due to difficulties in their procurement and isolation. Using a custom sequencing method, we explored the expression of seven sncRNA species (miRNA, piRNA, rRNA, scaRNA, snRNA, snoRNA, tRNA) from whole chorionic villi and four major sample-matched FACS-sorted cell type (cytotrophoblast, stromal, endothelial, Hofbauer) samples from 9 first trimester and 17 term placentas. After normalization for technical variables, samples clustered primarily by cell type lineage. No sncRNAs were uniquely expressed by cell type, however, mean expression differed by cell type for 115 sncRNAs. Known placentally-expressed sncRNAs showed differing expression by cell type and trimester. Expression of few sncRNAs varied by sex. Lastly, sample-matched sncRNA expression and DNA methylation correlation was not significant, although high correlation (> R^2^ ± 0.6) was observed for some sncRNA-CpG pairs. This study represents the first exploration of the sncRNA transcriptome of bulk placental villi and placental cell types, informing about the expression and regulatory patterns underlying human placental development.

## INTRODUCTION

Gene expression and cellular differentiation in the human placenta are dynamically regulated to ensure maintenance and progression of a healthy pregnancy. This temporary, highly complex organ facilitates all communication between the mother and fetus, and aberrant functioning can lead to severe consequences in both mother and baby. The human placenta is derived from the conceptus and is thus, in most cases, genetically identical to the fetus. It is anchored to the maternal uterus and is connected to the fetus by the umbilical cord; it serves as the interface for nutrient, gas, and waste exchange between the mother and fetus. Even though the placenta is invaluable in its role in sustaining life, it still remains under-investigated^1^.

The bulk of the human placenta consists of the chorionic villi (CV)^2^, which are branched, finger-like projections arising from the chorionic plate (the fetal side of the placenta). The CV are composed of several placental cell types, each with their own distinct developmental origins and functions^3^. The trophectoderm layer of the blastocyst differentitares into cytotrophoblast (CTB) cells, which form the inner lining of the CV. The outer layer of the CV (which are in direct contact with the maternal blood) arises from the fusion of these CTBs into a multi-nucleated synctium, and are termed as syncytiotrophoblasts (SCT) – the major cell type component of the CV^4^. CTBs proliferate through the SCT layer to form CTB columns, attaching the CV to the maternal decidua. At the ends of these columns, CTBs further differentiate into extravillous trophoblasts (EVT), which invade and remodel the maternal spiral arteries, facilitating maternal blood flow into intervillous space (IVS). The chorionic villous core contains mainly placental stromal cells (SC), endothelial cells (EC), and Hofbauer cells (HBC). The SCs are primarily fibroblast cells which maintain structural morphology and tissue homeostasis. ECs, which line the placental capillaries and are involved in cell-cell communication, play an essential role in the expansion of the placental vasculature and control blood flow. HBCs are M2-like placental macrophages derived from fetal monocytes that have several functions in the placenta including promoting vasculogenesis, angiogenesis, immune regulation, and transfer of ions and serum proteins^5^. Functional coordination of these various placental cell types is vital for proper structural maintenance, cellular communication, immune response, and vascular remodelling in the placenta.

Regulation of gene expression is governed by numerous factors, and small non-coding RNAs (sncRNAs) are one such post-transcriptional group of regulators. SncRNAs typically function by recruiting and binding to Argonaute and other proteins^6^ to form the RNA-induced silencing complex (RISC)^7^, leading to RNA interference (RNAi) via repression of messenger RNA (mRNA), modifications on ribosomal RNA (rRNA), and even sponging of other ncRNAs^8^. Several species of sncRNAs have been discovered, with each species designated by their transcription origination and the factors that they regulate. NcRNAs are transcribed genome-wide in all organs, and show moderate conservation across various species^9^. Due to their smaller size, sncRNAs are relatively stable and therefore less prone to degradation after tissue sampling than mRNA, and have thus been proposed to be more reliable biomarkers of health^10^.

The most widely studied sncRNA species are microRNAs (miRNAs), which are the smallest sncRNAs in length spanning 19–22 nucleotides, and known to bind to and repress mRNA through the RISC. Several studies have investigated miRNA expression in placental pathologies^11–14^, particularly for two placentally-expressed imprinted miRNA clusters on chromosome 14 and chromosome 19^15,16^. miRNAs can bind mRNAs with imperfect complementarity at all nucleotide positions other that at the miRNA seed sequence (nucleotides 2–8), meaning that a single miRNA targets several different mRNAs^17^. Piwi-interacting RNAs (piRNAs) primarily suppress repetitive elements and are critically important in mammalian sperm cells^18^, and we previously observed that their expression profiles are unique in the placenta relative to other organs^19^. The biogenesis of ribosomal RNA (rRNA) subunits, which interact with transfer RNA (tRNA) to translate mRNA into protein, is complex – erroneous rRNA sequences have been suggested to generate antisense sequences which may interfere with translation of normal rRNA itself^20^. Small nuclear RNAs (snRNAs) and small nucleolar RNAs (snoRNAs) modify pre-mRNA processing^21,22^. Finally, tRNAs are thought to engage with mRNA, along with speculated roles in regulating cell death^23,24^. While we have previously described the overarching expression landscape of snRNAs, snoRNAs, and tRNAs in the human placenta^25^, in-depth characterization of bulk placental tissue and placental cell type sncRNAs is still limited^26–29^.

To date, sncRNA studies in the placenta have focused on either bulk placental CV tissue or on the investigation of miRNAs, and in limited cell types (usually CTBs)^30,31^. Bulk tissue profiling gives an approximate measure of the average of all cell types, but the proportions of each placental cell type in the sampled tissue may bias the observed overall expression, and can output an inaccurate representation of the expression landscape^32–35^. With all placental cell types performing their own distinct roles in the growth and functioning of the placenta, elucidating the cell-specific sncRNA profiles would be an invaluable resource.

To advance our understanding of the factors that regulate gene expression in placental cell types, we explored the sncRNA transcriptome of four major fluorescence activated cell (FAC)-sorted placental cell types - CTBs, SCs, ECs, and HBCs and their matched bulk villi samples, in first trimester and term placental samples. We assessed the differences and similarities in the expression of sncRNA species within these cell types, identified how expression varies in association with biological variables such as gestational age, trimester, and sex, and assessed the correlation of sncRNA expression with sample-matched DNA methylation data. To our knowledge, this is the first exploratory resource of placental cell-specific sncRNAs, and our findings highlight the importance of considering the contributions of placental cell types when assessing bulk placental tissue.

## METHODS

### Sample acquisition and processing

Placental tissue samples were obtained with approval from The University of British Columbia / Children’s and Women’s Health Centre of British Columbia Research Ethics Board (H16–02280) as previously described^36^. All experiments were performed in accordance with relevant guidelines and regulations. In brief, for the 17 term (36–40 weeks) samples, pregnant individuals scheduled for a C-section at > 36 weeks gestation with a singleton pregnancy, and no pregnancy complications were recruited. Samples and data were collected by the BC Children’s Hospital BioBank (Vancouver, BC). The British Columbia Children’s Hospital BioBank (BCCHB) is an institutional biobank that collects samples and data from both children and women at BC Children’s and Women’s Hospitals and Health Centres for future, ethically approved research. The BCCHB also supports local research projects through several services such as consenting, sample processing, and sample storage. Additionally, 9 de-identified first trimester (6–13 weeks) placental samples from elective terminations were obtained, for which no gross pathologies were detected. All samples were confirmed to be chromosomally normal using copy number variant (CNV) calling on the Illumina Infinium MethylationEPIC array^37^.

For bulk CV term samples (*n* = 17), three to four sites were sampled from below the surface of the fetal-facing side of the placental disc to avoid maternal contamination. These samples were preserved in RNAlater and pooled for processing. The first trimester placentas (*n* = 9) are physically smaller in size to allow for multiple sites being sampled and pooled together, therefore a small singular tissue sample was preserved in RNAlater for all first trimester placentas. From each of the 26 pooled or single CV samples, 30 mg of tissue was processed, and excess RNAlater was removed by blotting. Samples were homogenized in the Next Advance Bullet Blender Tissue Homogenizer (Next Advance, USA), using 3.2 mm stainless steel beads (Next Advance, USA) with 1 ml of TRIzol reagent (ThermoFisher Scientific, USA). Samples were incubated for five minutes at room temperature (RT), and then centrifuged at 7000 rpm for three minutes. 200 ml of chloroform (ThermoFisher Scientific, USA) was added to the supernatant, which was thoroughly mixed by inversion, and incubated for five minutes at RT. Samples were centrifuged at 4°C at 9000 rpm for 20 minutes, and 500 μl of isopropanol (ThermoFisher Scientific, USA) was added to the aqueous phase mixed by inversion, and incubated for 10 minutes at RT. Samples were centrifuged at 4°C at 9000 rpm for 15 minutes. Supernatant was discarded and the RNA pellet was washed in 75% ethanol by gentle inversion. Samples were centrifuged at 4°C at 9000 rpm for 10 minutes. Supernatant was discarded, and the RNA pellet was air-dried for five minutes at RT. RNA was eluted in 50 μl of ultrapure distilled RNAse/DNAse free water (Gibco-LifeTechnologies, USA). Genomic DNA removal was carried out using the RNase-Free DNase Set (Qiagen, Germany). RNA concentration was measured on a Nanodrop 2000 (ThermoFisher Scientific, USA), and RNA quality was assayed on an Agilent Bioanalyzer 2100 (Agilent, USA). CV samples were extracted by two separate people using the same protocol (extraction type 3, listed in Results)

Sample processing and cell isolation was described previously^36^. Briefly, for the FAC-sorted placental cells (*n* = 104 representing four cell types per each of the 26 placentas), fresh CV samples were dissected and processed into a single-cell suspension (as described^36^), and then stained for various markers to be sorted by FACS using the BC Children’s Hospital Research Institute FACS Core Equipment. Dead cells and red blood cells were first gated out using 7AAD-A + and CD235a+, respectively. Specific cell populations were then identified using various surface markers: CTBs as CD34−CD45−CD14−EGFR+, SCs as CD34−CD45−CD14−EGFR−, ECs as CD34 + CD45−, and HBCs as CD14 + CD34−. Cells were collected directly in DNA/RNA Shield 2x concentrate or Zymo RNA Lysis Buffer, stored at −80°C, and total RNA was extracted from each sorted-cell population using the ZYMO Quick-RNA MiniPrep kit according to manufacturer’s instructions (extraction type 1, listed in Results). Two samples were extracted using TRIzol (ThermoFisher Scientific, USA) (extraction type 2, listed in Results). All samples were DNAsed using the QIAGEN RNase-Free DNase Set before sequencing (QIAGEN, Germany).

As an initial quality control step, maternal contamination of all bulk and sorted cell samples was assessed as described previously^36^. Samples predicted to have > 35% contamination based on Illumina DNA methylation array data and those with poor RNA concentration (< 1 ng/ul) were excluded. 28 of the 130 samples (130 samples = 26 CV + 104 sorted cells) showing high maternal contamination were excluded, of which the majority were first trimester (excluded: 8/26 CTB, 0/26 SC, 9/26 EC, 9/26 HBC, 2/26 CV), retaining 102 samples (130 total samples minus 28 high maternal contamination samples) for RNA sequencing (Table 1).

### RNA sequencing

RNA sequencing was performed at the Canada’s Michael Smith Genome Sciences Centre (GSC) in Vancouver, Canada. The sequence read length profile reflects the size distribution of the isolated RNA **(Supplementary Fig. 1)**, with the protocol being specific for miRNAs (fragments between 18–24 nucleotides in length). However, partial matches to other longer sncRNA species were also possible. Samples were sequenced based on a low-input RNA protocol described by Hagemann-Jensen et al^38^. Briefly, 5.8S rRNA was masked during adapter ligation by a complementary oligonucleotide, while miRNAs underwent 3′ adapter ligation, digestion of unligated adapters, and 5′ adapter ligation sequentially. The 5′ adapter contains a unique molecular identifier (UMI), followed by two nucleotides (CA) prior to the miRNA sequence. Upon conversion to cDNA by reverse transcription, the cDNA was amplified by PCR to include the sequences required for Illumina cluster generation and sample indexing developed by the GSC. The number of PCR cycles was dependent on the amount of input RNA; samples with low and high input were subjected to 20 and 15 cycles of PCR, respectively. Quality of libraries was assessed using the Agilent Bioanalyzer 2100 HS DNA chip Assay, (Agilent, USA) after which size selection for the miRNA fraction was conducted using the Blue Pippin 3% agarose gel cassette (Sage Science Inc., USA). Samples were pooled for single-end 75 bp (SE75) sequencing on the Illumina NextSeq 500 (Illumina, USA) on two plates. Ten samples were sequenced initially (Batch 1) to estimate the minimum sample concentration required for subsequent sequencing (10ng/ul for sorted cells, 50 ng/μl for CV), and for the remaining 92 samples (Batch 2), 4 pools of 24 libraries each were aggregated and sequenced.

### Data processing and analysis

Raw sequencing reads were trimmed to remove the 10 nucleotide UMI consisting of the eight nucleotides and CA dinucleotide from the 5’ end, the 20 nucleotide Illumina TruSeq 3’ adapter sequence (TGGAATTCTCGGGTGCCAAG), and all nucleotides after the 3’ adapter. FASTQC[http://www.bioinformatics.babraham.ac.uk/projects/fastqc/] was used to assess sequencing quality metrics of samples. Trimmed reads were uploaded to the online miRMaster2 (v 2.0.0) tool**[33872372]** to discard sequences with less than 17 nucleotides, perform quality ltering using Phred > = 20 over a four-nucleotide sliding window, and to align and count raw reads relative to the human GRCh37 reference genome using STAR**[23104886]**. miRMaster2 utilizes sequence data from miRBase (22.1), Ensembl ncRNA (v 100), RNACentral piRNA (v 15), GtRNAdb (v 18.1), and NONCODE (v 5), and trimmed reads were aligned for seven sncRNA species using these databases: microRNAs (miRNAs), Piwi-interacting RNAs (piRNAs), ribosomal RNAs (rRNAs), small Cajal body-specific RNAs (scaRNAs), small nuclear RNAs (snRNAs), small nucleolar RNAs (snoRNAs), and transfer RNAs (tRNAs). Manual parameters for alignment were: 3’ Adapter = TGGAATTCTCGGGTGCCAAGTCGACGTACGATC, 5’ Barcode length = 0 (as already trimmed beforehand), Adapter barcode length = 0 (as already trimmed beforehand), UMI length = 0 (as already trimmed beforehand), Minimum read length = 17 (minimum length for miRNAs), Alignment tool = STAR. All other parameters selected were default settings (after consulting with the author of the software that the default parameters were suitable for use with the specific sequencing method that this study used).

miRMaster2^39^ has been trained specifically for the alignment and annotation of non-coding RNAs and includes 72 sucessive steps in aligning reads to the genome, with extensive user-defined input parameters avialable. All non-coding RNAs thus had high confidence of alignment and subsequent read counts, with the known caveat that higher read counts can lead to a higher overall percentage of reads aligned. For sncRNAs other than miRNAs which have read lengths of > 22, those with perfect partial matches are also aligned and annotated by miRMaster2. These sncRNAs were explored with caution as the sequencing protocol for this study was specific towards miRNAs. Longer sncRNAs inherently have a greater probability of alignment (as longer genes have several regions that a read can potential map to); miRMaster counts each read alignement as one count, however, this bias is present across all samples for longer sncRNAs, and thus is effectively nulli ed between cross-sample comparisons (as confirmed in personal correspondence with the author of the software). The non-normalized, raw count matrices for the above seven sncRNA species were downloaded to be processed for further analysis.

All analyses were carried out in R (v4.3.1)^40^. To assess sequencing quality, a variable termed ‘sample quality’ was constructed by combining the wo sequencing metrics: the total number of reads sequenced (greater or less than 1 one million) and percent of reads mapping to miRNAs, as this protocol was geared towards selecting for miRNAs. Samples with either i.) total number of reads less than one million or ii.) less than 1% of reads mapping to miRNAs were flagged as ‘red’; 1–30% of total reads mapping to miRNAs as ‘yellow’; either i.) total number of reads more than one million or ii.) more than 30% of reads mapping to miRNAs as ‘green’. In cases where the total reads or total reads mapped to miRNAs did not have the same sample quality colour, the lower sample quality was assigned. The numerical cutoffs for each category were based on the expected sequencing quality as provided by the GSC. Five samples with sample quality designated ‘red’, two sorted-cell samples extracted by a different extraction method than the rest (extraction type 2 as mentioned above i.e., TRIzol), two EC samples that clustered with the CV samples along with having a ‘yellow’ sample quality, and one first trimester CV sample which had both its matched sorted-cell samples (SC and EC) assigned as outlier samples were removed from further processing (total samples removed = 10). Hence, the total samples that passed quality control were 92 of the original 102, as described in Table 1.

Data was batch-corrected using COMBAT-seq^41^ for the two sequencing batches (Batch 1 and 2), normalized using the Relative Log Expression (RLE) method, and counts per million scaled using the ‘edgeR’ (v4.0.16)^42^ and ‘DESeq2’ (v1.42.1)^43^ packages. Principal Component Analysis (PCA) was conducted using the prcomp function from the base R ‘stats’ package (v4.3.1)^40^ and the strength of association between metadata variables and principal components was estimated using linear models implemented with the plomics package (v 0.2.0)^44^

Sequence data of all samples were split by cell type (CTB, SC, EC, HBC) for all sncRNAs that passed quality control. sncRNAs that had a normalized count > = 2 in at least 75% of term samples per cell type were retained. The mean expression of each of these sncRNAs was compared amongst all four cell types using the Kruksal-Wallis test. If the mean expression of a sncRNA in one cell type was significantly different than in all other cell types at a Benjamini-Hochberg (BH) corrected *p*-value < = 0.05, the sncRNA was designated as cell type-associated. Several databases and software tools were used to mine information about sncRNA species including miRBase v22.1^45^, piRBase v3.0^46^, RNACentral v22^47^, GeneCards^48^ and GeneCaRNA^49^, HUGO Gene Nomenclature Committee (HGNC)^50^, GtRNAdb^51^, UCSC Genome Browser^52^, and Ensembl^53^, as each database provides non-overlapping information.

Linear regression implemented with the ‘limma’ package^54^ was used to identify sncRNAs that showed differing expression by sex. Sex differences in expression were assessed only in the term samples and separately within each cell type for sncRNAs on a) all autosomes and b) only the X and Y sex chromosomes, using the formula:

NormalizedReads~Sex+Plate+ExtractionType+ε

where, Plate stands for the physical sequencing plate ID. BH multiple testing correction was applied.

The reason for not using linear regression to observe cell type expression differences was the overfitting of linear regression models, due to the small sample size. For sex, as the independent variable was binary (XX or XY), and the confounding variables were categorical, linear regression estimates showed higher confidence.

Gene and pathway enrichment for sncRNAs of interest was carried out using the Molecular Signatures Database (MSigDB)^55,56^, pathDIP v5.0.32.3^57^, DIANA-miRPath v4.0^58^, and miRPathDB v2.0^59^, where all databases provide different types of miRNA-to-function predictions.

### sncRNA expression correlation with DNA methylation

Cell type sample-matched DNA methylation normalized beta (β) values were downloaded for the samples in this study from the GEO accession GSE159526 dataset. Non-variable CpG sites were removed. CpGs up to 10 kilobases (kb) upstream or downstream of sncRNA sequences that passed quality control in the first part of this study were selected: 586 CpG sites passed this criterion, corresponding to 77 sncRNAs, with some sncRNAs having multiple probes located within the 20kb window. Multi-omics integration tools require data to be split into training (70%) and testing (30%) datasets, however, as the number of term samples per cell type were less than 20, we applied Spearman Rank Correlation to test the relationship between sncRNA expression and DNA methylation beta values. A total of 702 sncRNA-CpG pairs were assessed for correlation, and adjusted for multiple testing correction using the BH method at *p*-value < = 0.05.

## RESULTS

### Landscape of placentally-expressed sncRNAs

As the first exploratory study of the sncRNA transcriptome of sample-matched placental cells and chorionic villi (CV), we firstly undertook an in-depth examination of sample quality (**Supplementary Methods**). To then explore sncRNAs that may have broad roles in cell and placental maintenance, sncRNAs that were consistently highly expressed in all term samples in all four cell types at RPM > = 1000 were examined. As the cell-sorted samples were lower quality, a threshold of 1000 reads was chosen instead of the commonly-used threshold of 10000 reads in similar profiling studies.188 sncRNAs (1% of the 9485 sncRNAs that passed quality control) satisfied this criterion; of these, 133 also had RPM > = 1000 in bulk CV (16 miRNAs, 32 piRNA, 7 rRNA, 10 scaRNA, 24 snoRNA, 24 snRNA, and 20 tRNA **[Supplementary Table 1**, **Supplementary Fig. 2]**). Expression of these 133 sncRNAs was not consistent across cell types - when expression for each individual sncRNA was compared between the four cell types, CTB and SC showed the most similar expression (Wilcoxon-Rank-Sum FDR > 0.05 for 100/133 sncRNAs), whereas CTB and HBC had the most dissimilar expression (FDR > 0.05 for 52/133). Interestingly, when only the 16 miRNAs were considered, 7/16 miRNAs had similar expression (FDR > 0.05) in CTB and EC, with SC and HBC having the most dissimilar (2/16 with FDR > 0.05). Pathway enrichment of the 16 miRNAs highlighted several critical developmental signalling and homeostasis pathways such as fatty acid biosynthesis, proteoglycan functioning, and Hippo signalling (FDR < = 0.05 using the DIANA-miRPath 3.0 software’s DIANA-microT)^60^ **(Supplementary Table 2)**, and GO annotations output were for angiogenesis, cell migration, and apoptosis. Surprisingly, hierarchial clustering of these 16 miRNAs (**Supplementary Fig. 2**) grouped samples by cell type, further underscoring that even for fundamental functional pathways, cell types can exhibit significant expression differences - and in the context of this study, differing levels of gene regulation that can be exerted.

**Figure 1:**
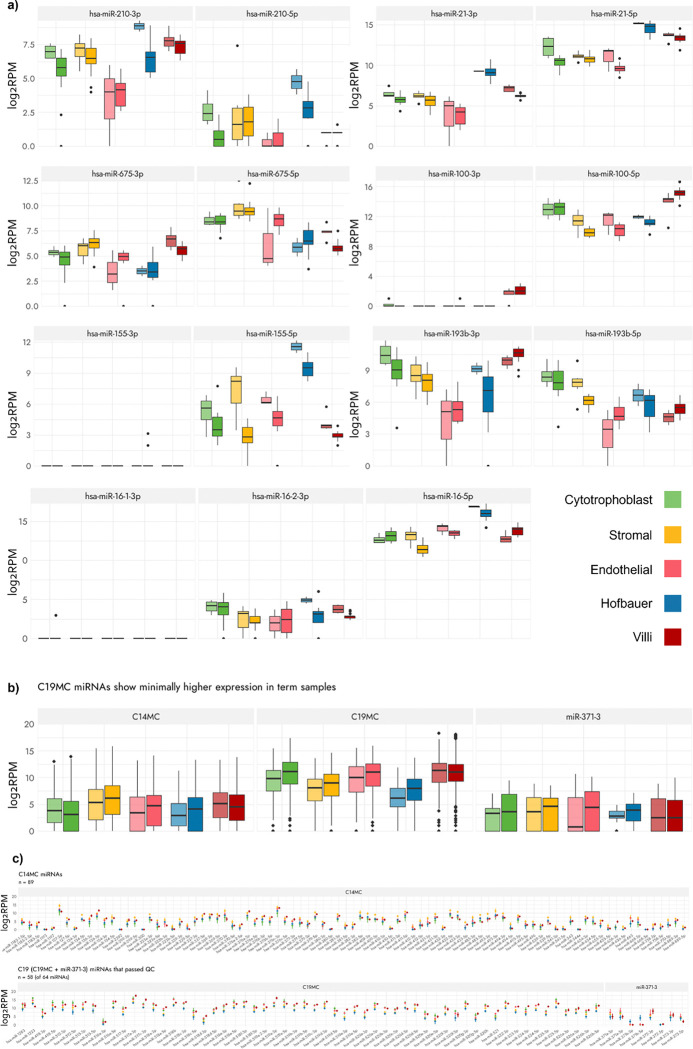
Expression of known placentally-expressed miRNAs. All reads are per million. **a)** The expression of 7 miRNAs known to be associated with placental / pregnancy disorders, where the lighter coloured boxplots on the left are first trimester samples, and the darker colour boxplots are term samples. **b)** Combined expression of the 89 C14MC, 59 C19MC, and 8 miR-371–3 cluster miRNAs in first trimester and term samples in the different cell types and matched whole villi. **c)** Cell-specific and CV expression of each individual miRNA in the C14MC, C19MC, and miR-371–3 clusters in term placentas. The dots represent the mean, and the vertical lines on either side of the dot represent the standard deviation.

We next studied the expression of sncRNA species previously reported to be expressed and relevant in the human placenta. To date, published studies examining roles of sncRNA species in the placenta have been largely limited to the study of miRNAs^12,26,31^; for example, mature miRNAs (most commonly designated with a suffix of either − 3p or −5p after the name of the precursor miRNA) of the precursors hsa-miR-100, hsa-miR-16, hsa-miR-155, hsa-miR-193b, hsa-miR-21, hsa-miR-210, and hsa-miR-675 have been previously identified for showing altered expression in placentas from pregnancies of preeclampsia (PE), fetal growth restriction (FGR), and/or intrauterine growth restriction (IUGR)^61,62^. Indeed, we found that all these miRNA species showed expression differences between cell types, and even between first trimester and term samples for the same cell type - except for hsa-miR-100–3p, hsa-miR-155–3p, and hsa-miR-16–3p, which were largely undetected in our samples (Fig. 1a).

Three well-studied imprinted miRNA clusters located on chromosome 14 (C14MC), chromosome 19 (C19MC), and the miR-371–3 cluster have been implicated in placental functioning^31,63^. C14MC miRNAs are most highly expressed during the first trimester of pregnancy, while C19MC miRNAs have the opposite pattern, with expression levels peaking in the later stages of pregnancy. Among the 161 total miRNAs lying within these clusters, we found that all 89 C14MC miRNAs, 59 of 64 C19MC miRNAs, and all 8 miRNAs from the miR-371–3 cluster had non-zero expression in most cell types. The exceptions were hsa-miR-371b-3p which had no expression in CTBs and SCs, hsa-miR-371b-5p with zero expression in CTBs, and hsa-miR-372–5p with expression only in HBCs. Interestingly, while the expected expression pattern (slightly higher in term samples compared to the first trimester) was observed for the C19MC miRNAs, the mean expression levels of the C14MC miRNAs showed no difference between trimesters (Fig. 1b); this discrepancy may be attributed to the comparatively lower sample numbers and potentially reduced sample quality of the first trimester samples relative to term. Moreover, when we examined the expression levels per miRNA individually within these clusters (Fig. 1c), all 89 C14MC had the highest expression in SCs (Wilcoxon-Rank-Sum FDR < 0.05). Conversely, for the C19MC miRNAs and the miR-371–2 cluster miRNAs, CTBs and ECs exhibited the highest expression, respectively. For all C19MC miRNAs, HBCs also showed the lowest expression. This difference in expression levels within the cell types and their comparison to the CV expression underscores the importance of cell type consideration, also highlighting how expression within these different cell types changes with gestation.

The average expression across all cell types for the miRNA clusters roughly matched the average expression of the CV samples. When expression of all cell types was combined and compared with that of villi, no statistically significant difference in expression was observed overall (hsa-hsa-miR-100–3p and hsa-hsa-miR-100–5p being the exception, where expression in CV was higher). CTB expression did not match closely with that of CV, but it should be noted that the major component of the CV samples are STBs, and that we were not able to isolate these cells by FACS due to their presence as a continuous syncytium. It is possible that STBs have a very different sncRNA profile than their CTB progenitors, despite their DNAm profile being quite similar. This further highlights the need for and importance of considering cell composition when studying the placenta, as sampled placental tissue may in fact not represent the true expression landscape.

### Placental cell type-associated sncRNA profiles

To determine if the placental sorted-cells had cell type-specific expression of sncRNAs, we investigated each cell type independently. As we had fewer first trimester samples (*n* < 8 for each cell type), and to maintain consistency, we restricted this analysis to only term samples (*n* = 71).

**Figure 2:**
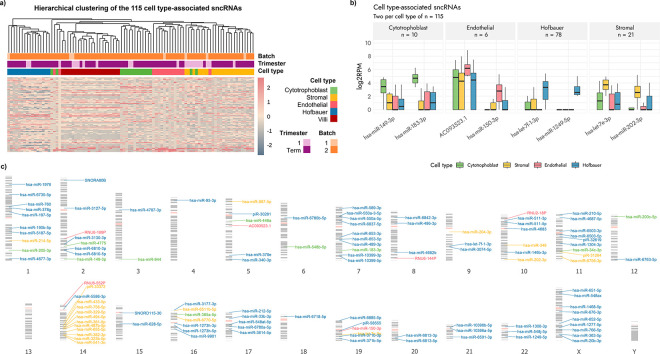
Cell type-associated sncRNA expression. **a)**Clustering heatmap (Z-scores of log2-transformed normalized expression counts) of the 115 cell type-associated sncRNAs. **b)**Expression of two examples each of cell type-associated sncRNAs detected per cell type. *n* represents the number of sncRNAs designated as cell type-associated for that specific cell type. **c)** Chromosomal location of each of the 115 cell type-associated sncRNAs.

No sncRNAs showed unique expression in a single cell type. We thus sought to investigate cell type-associated expression by considering sncRNAs with a read count of >=2 in at least 75% of term samples (i.e., expressed in at least 9/12 CTB = 1,836 sncRNAs; 12/16 SC = 1,879 sncRNAs; 9/12 EC = 1,825 sncRNA; 10/14 HBC = 2,081 sncRNAs) for each cell type were considered. A proportion of the above selected sncRNAs, 115 sncRNAs, showed significantly different mean expression in one specific cell type when compared to the mean expression of the other cell types (Kruskal-Wallis BH *p*-value <= 0.05), and were classified as cell type-associated sncRNAs (Table 2, Figure 2a and 2b, Supplementary Table 3).

These cell type-associated sncRNAs were spread across the genome (Table 2, Figure 2c). Surprisingly, none of the 103 cell type-associated miRNAs we identified belonged to either the placental-associated miRNA clusters on chromosomes 14 or 19. Of the 103 cell type-associated miRNAs, 39 have previously been reported in the literature as having some relation (pathogenic or not) with the human placenta; the remaining 64 miRNAs and 12 non-miRNAs are, to our knowledge, novel associations **(Supplementary Table 4)**.

HBC sncRNA expression was the most distinct as compared to all other cell types, attesting to the unique embryonic origin of HBCs as compared to the other cell types (Figure 2a). Seven of the 78 HBC-associated miRNAs mapped to the X chromosome (hsa-miR-20b-3p, hsa-miR-1277–5p, hsa-miR-652–5p, hsa-miR-676–3p, hsa-miR-1468–5p, hsa-miR-548ax, hsa-miR-651–5p), these were the only cell-associated sncRNAs were detected on the X chromosome (Figure 2c). Given their presence on the X chromosome, we also compared the expression between sexes (placentas from male and female fetuses) for these seven miRNAs, however none showed significant expression differences by sex.

### Shared miRNAs between cytotrophoblast and cancer

**Figure 3:**
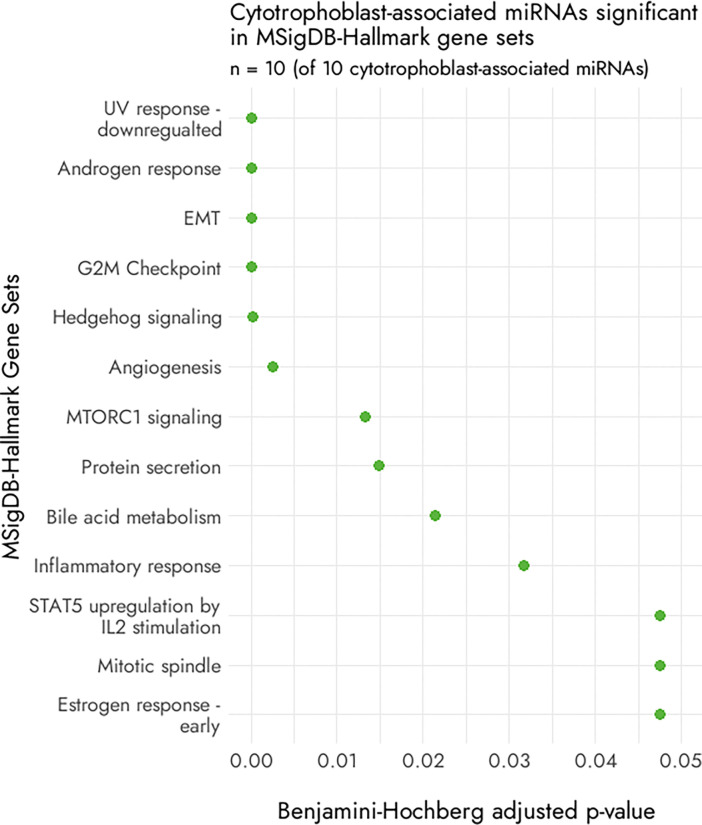
Pathways predicted for the 10 cytotrophoblast cell-associated miRNAs. Gene sets for which all 10 cytotrophoblast cell-associated miRNAs were significant at a BH *p*-value <= 0.05.

Due to the shared invasive and stem-like properties of CTBs and cancer cells, we examined the 10 CTB-associated sncRNA (all miRNAs) for their relevance to cancer. A PubMed search (search terms: [miRNA] AND cancer) for the 10 CTB-associated miRNAs (hsa-miR-149–3p, hsa-miR-183–3p, hsa-miR-200c-5p, hsa-miR-205–3p, hsa-miR-34c-3p, hsa-miR-365a-5p, hsa-miR-449a, hsa-miR-4775, hsa-miR-548b-5p, has-miR-944) revealed that all have previously been reported for association with several cancer types, most commonly lung, breast, and colorectal cancer. The term ‘epithelial-mesenchymal transition’ was also reported in either the abstract or the main text of the publications for six of the 10 miRNAs. All 10 miRNAs were also significant (FDR < = 0.05) for being included in 13 of the 50 Hallmark Gene Sets within the Molecular Signatures Database (MSigDB)^55,56^, a database that contains manually curated gene set lists for several disease conditions (Fig. 3).

Expression for these 10 CTB-associated miRNAs was also compared between tumour and normal samples within The Cancer Genome Atlas (TCGA) cohorts, and several cohorts showed expression differences between malignant and non-malignant samples for these miRNAs (**Supplementary Fig. 3**); expression of oncogenic miRNAs is generally upregulated in cancers, where high expression of these oncomirs leads to suppression of key target tumour suppressor genes^64^.

### Sex-specific profiles of placental cell types

Sex chromosome complement (XX or XY) can also contribute towards inter-individual variability, contributing towards expression differences in genes located on both the autosomes as well as the sex chromosomes^65–67^. We investigated whether placental sex was associated with sncRNA expression in term placental cell type samples.

**Figure 4:**
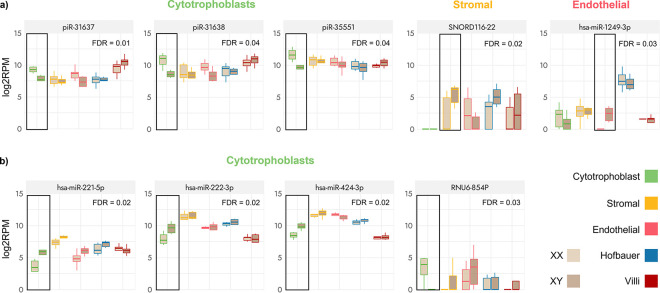
SncRNAs showing variable expression by sex in term sorted-cell placental samples. **a)** Autosomal sncRNAs which showed differing expression by sex in each cell type. The lighter coloured boxplots on the left are XX samples and the darker coloured boxplots are XY samples. **b)** X-linked sncRNAs with differing expression by sex in cytotrophoblast sorted-cell samples.

When only CV samples were considered, no autosomal or sex-linked sncRNAs showed significantly differing expression by sex. Within the sorted-cell samples, at a BH *p*-value < = 0.05, three piRNAs (piR-31637, piR-31638, piR-35551) showed higher expression in XX as compared to XY CTB samples, while higher expression was observed for one snoRNA (SNORD116–22) in XY SC samples and one miRNA (hsa-miR-1249–3p) in XY EC samples (Fig. 4a) on the autosomes. Hsa-miR-1249–3p has previously been identified as contributing towards angiogenesis^68^.

When only the X and Y chromosomes were considered, three X-linked miRNAs (hsa-miR-221–5p, has-miR-222–3p, hsa-miR-424–3p) had higher expression in XY samples as compared to XX and one snoRNA (RNU6–854P) had higher expression in XX CTB samples (BH p-value < = 0.05) (Fig. 4b). Of the three X-linked miRNAs, hsa-miR-221 and hsa-miR-424 have previously been reported in the context of placental and/or pregnancy disorders^69–71^. None of the four Y-linked miRNAs (hsa-miR-3690–2, hsa-miR-6089–2, hsa-miR-9985, hsa-miR-12120) that had passed quality control showed differing expression by sex.

### Assessing correlation between cell type-associated sncRNAs and DNA methylation

We previously comprehensively cataloged the DNA methylation profiles of the same sorted-cell samples used in this study with the Illumina MethylationEPIC array^36^. DNA methylation, similar to sncRNAs, plays a pivotal role in the epigenetic regulation of gene expression. In many instances, these two regulatory mechanisms work synergistically to influence downstream gene expression^72,73^.

To assess any correlation that may exist between sncRNA expression levels and DNA methylation, previously processed sample-matched DNA methylation data was considered^36^. The expression of the 115 cell type-associated sncRNAs was measured against CpGs that fell within 10Kb upstream of the 5’ end and 10Kb downstream of the 3’ end; this basepair window differs by study^74,75^, and we chose 10kb to assess any cis-interactions. 400 CpG probes fell within these 20Kb windows for 77 of the 115 cell type-associated sncRNAs, and for some sncRNAs there was more than one sncRNA-CpG pair (there were multiple CpGs present within the 20Kb window for 63 miRNAs). When normalized sncRNA RPM expression was compared with normalized DNA methylation beta values, no sncRNA-CpG pairs were significantly correlated at a BH *p*-value < = 0.05 in term samples. Albeit not reaching the established threshold of multiple-testing correction significance, we did observe high correlation (R^2^ > 0.6 or R^2^ < −0.6) between six CTB, two SC, and two HBC sncRNA-CpG pairs (*n* = 10) (**Supplementary Fig. 4, Supplementary Table 5**).

When only term CV samples were considered, one sncRNA-CpG pair had R^2^ > 0.8 (BH *p*-value = 0.65): hsa-miR-512–3p (intergenic) and cg27369447 (3’ UTR of *ZNF665*) on chromosome 19 (**Supplementary Fig. 4**). Hsa-miR-512–3p belongs to the placental-exclusive, paternally-expressed C19MC^76,77^. When CpGs and miRNAs lying only within the C14MC (14q32.31) and C19MC (19q13.42) regions were separately examined in term villi samples, only one pair within C19MC passed the significance threshold (BH *p*-value = 0.00) and had R^2^ < 0.8 - hsa-miR-523–3p and cg24815529 (**Supplementary Fig. 4**). Hsa-miR-523–3p has been previously shown to be downregulated in PE^78^. Lastly, despite not reaching multiple testing significance, 28 miRNA-CpG pairs on 14q32.31 and 10 pairs on 19q13.42 had high correlation values (R^2^ > 0.8 or R^2^ < −0.8) (**Supplementary Table 5**).

## DISCUSSION

We characterized the small non-coding RNA profiles of four major placental cell types (cytotrophoblasts, stromal cells, endothelial cells, and Hofbauer cells) and their matching chorionic villi (CV) samples in first trimester and term placentas. To our knowledge, this is the first genome-wide exploratory profiling of placental cell-specific sncRNAs. A limitation of our study is that it did not investigate synctiotrophoblasts which represent the major cell type of the bulk placenta (due to the inability to sort this cell type by FACS as its composition of a continuous syncytium); however, we estimate the bulk tissue samples themselves to be representative of this particular cell type due to it’s high abundance in placental tissue, demonstrated in other studies^36,79^. While no sncRNAs showed unique expression in just one cell type, certain sncRNAs showed significantly different mean expression in one cell type as compared to the other cell types, which we termed as ‘cell type-associated sncRNAs’. These 115 cell type-associated sncRNAs were distributed across chromosomes, and differences in their expression profiles were also indicated by PCA and hierarchical clustering.

HBCs - macrophages that play a role in both immune response and angiogenesis^5,80^ - showed the highest number of cell type-associated sncRNAs (n = 78), and target prediction for these HBC-associated sncRNAs identified similar functional pathways **(Supplementary Fig. 5)**. Interestingly, the expression levels of these 78 HBC-associated sncRNAs was distinct from the other cell types, indicative of their unique developmental lineage, and HBC samples also grouped in their own top-level cluster separate from CVs, CTBs, SCs and ECs (Fig. 2a).

The CTB-associated sncRNAs we identified were significantly enriched for cancer-related pathways; trophoblasts fundamentally invade into the maternal endometrium to anchor the placenta and establish blood flow, and this invasive and proliferative nature is often likened to cancer cells^81^. These 10 CTB-associated sncRNAs, which were all miRNAs, have been previously reported to be differentially expressed in several placental disorders^82,83^ and several cancer subtypes^84–87^, providing further support in the shared characteristics of placental growth and cancer.

ECs and SCs are both derived from the extraembryonic mesoderm and have shown the most similar DNA methylation patterns than other cell types^36^, we did not find sncRNA expression to be more closely related in these relative to the other cell types. SCs are known to be important in structural maintenance and immunomodulation, and the predicted biological pathways for the 21 SC-associated sncRNAs we discovered reported the above functions **(Supplementary Fig. 5)**, with pathways such as *MTORC1* and cell cycle checkpoint signalling^88,89^. Interestingly, half (11/21) of the SC-associated sncRNAs were located on chromosome 14, with the majority of these being intergenic (9/11). The six EC-associated sncRNAs (here, sampled from small chorionic villi vessels that are found in the mesenchymal core) were enriched for pathways significant for cell communication and signalling (**Supplementary Fig. 5)**, also consistent with previous findings^90,91^.

Three known placental miRNA clusters, the imprinted C14MC, C19MC, and miR-371–3 cluster, each showed differing expression by cell type, and cell-specific expression of these miRNAs has previously been suggested^76,92^. In all 89 C14MC miRNAs, SCs had the highest expression, CTBs had higher expression for most of the 58 C19MC miRNAs, and ECs had the highest expression for the eight miRNAs belonging to the miR-371–3 cluster (the expression difference between these cell types and others was not substantial for them to be classified as cell type-associated, however). Suprisingly, even though the C19MC (19q13.41) and miR-371–3 (19q13.42) clusters only lie about 30kb apart, the cell-specific expression was distinct in these two clusters, as alluded in previous studies^92^. While the exact functions of these clusters is not fully known, their expression has markedly distinguished the placenta from other organs^93^, and has often observed to differ in cancer^94,95^. Altered levels of C19MC miRNAs have shown to impact trophoblast development^70,96,97,76^, and C14MC miRNAs have shown high expression in CV and chorionic plate-derived SCs as compared to decidual basal plate-derived cells^92^. Placental miRNAs are also known to enter the maternal bloodstream encased in extracellular vesicles (EVs)^98^, and C19MC miRNAs have been reported to be the predominant miRNAs in placenta-secreted (predominantly CTB-secreted) exosomes in maternal serum^99^. They may thus have potential utility as specialized serum biomarkers for pregnancy complications (such as gestational diabetes mellitus (GDM), gestational hypertension, PE, FGR) should their expression patterns be capable of distinguishing healthy from pathogenic samples associated with these conditions^100,101^.

We also compared the expression between XX and XY placentas, separately in the autosomes and sex chromosomes. Five autosomal sncRNAs (three piRNAs in CTBs, one snoRNA in SCs, and one miRNA in ECs) and four X-linked sncRNAs (three miRNAs and one snRNA, all in CTBs), had significantly differing expression between the sexes. That differences exist in placentas carrying male fetuses compared to female fetuses is known; on average, placentas from male fetuses weigh more and show higher expression of immune-related genes, and carrying male fetuses has an increased risk for placental complications^102^. While still limited, our results build on the evidence in expression differences observed between the sexes^103^.

While we did not find a strong correlation between sncRNA expression and DNA methylation profiles within our matched samples, crosstalk between the two features does occur^72,75,104^. The lower sample size and power of this study may have been a reason for none of the sncRNA-CpG pairs passing multiple-testing correction. Given the rarity of acquiring isolated placental cells, the use of instruments and methodologies that incorporate multi-omic measurements in future placental studies would allow for maximal data generation from these scarce tissues (for example, using technology such as the Oxford Nanopore MinION, which allows for sequencing and DNA methylation measurement simultaneously).

The novelty of this study is three-fold: first - we assessed the expression of sncRNAs in four major placental cell types, second - we concurrently examined expression in sample-matched placental cell and whole villi samples, and third - we profiled several understudied sncRNA species in the placenta. Our results show that inter-placental and inter-cell type variability exists for several sncRNAs (as show in Fig. 2a, Fig. 4a), in particular for known placental-expressed miRNAs. While studies of sncRNA species in one cell type (for example, miRNA expression in TBs) have been highly informative in discovering sncRNAs associated with specific conditions (such as hsa-miR-16, hsa-miR-21 and hsa-miR-210 in PE and hsa-miR-100b in IUGR^105,106^), the magnitude (level of expression) and direction of expression (over or under expression) of the sncRNA profiles between and within placental cell types has generally not been analyzable in the majority of studies. Furthermore, gene expression differences are also known to exist between placental and other extraembryonic tissues such as the umbilical cord, amnion, and decidua^107,108^, providing a future avenue of investigation.

A limitation of our study was the cell-sorting procedure involved several time-intensive processing steps which may have impacted cell quality (i.e., due to stress that the samples may have incurred). Also, the low overall sample yield after cell-sorting could have produced a higher number of artefact sequences. However, all sorted-cells were processed sequentially from the same sample, and care was taken to be consistent for all samples. To furthermore alleviate low sample quality, our designation of cell type-associated sncRNAs was based on non-stringent selection, in an effort to perform a generous profiling estimation. The lower number of first trimester and the non-matching number of samples for each cell type also led to unbalanced sample numbers, however we tried alleviating this by examining each cell type, and first trimester and term samples separately. The sequencing and alignment protocol was also geared towards miRNAs, and as such, all sncRNA species were analysed per species to ensure that species-dependant variability was controlled for. Lastly, a very low percentage of previous sncRNA associations have proven to be reproducible to date^28^ due to small sample sizes, varied processing procedures, the downstream effects of non-standardized genome alignment, data quality control, and analysis pipelines - we highlight how technical variables influence expression levels and the need in controlling for their effects for reliable expression profiling.

## CONCLUSION

To our knowledge, this is the first exploratory study of the small non-coding RNA transcriptome of the human placenta and its cell types. This study provides insight into the sncRNA profiles of four isolated placental cell types and whole chorionic villi in sample-matched placentas from both the first trimester and term samples. Our results summarize that certain sncRNAs are expressed more distinctly in one cell type in comparison to others, and build on the proposed functions of these cell types from previous studies. Overall, the profile of bulk placental villi was distinct from the individual cell types, underscoring that whole tissue expression is not representative of individual cell type expression. This data is a novel resource for furthering our understanding of placental cell type heterogeneity and its contributions to regulatory mechanisms in the healthy placenta.

## Figures and Tables

**Table 1:** Sample distribution. The total 92 sorted-cell and matching chorionic villi samples that passed quality control and were included for analysis. The numbers in parentheses are the total samples of the 102 samples originally sequenced.

	First Trimester	Term	Total
	Placentas = 8 (9)	Placentas = 17	Placentas = 25 (26)
	Samples = 21 (28)	Samples = 71 (74)	Samples = 92 (102)

**Sex**			

Female	5 (6)	9 (9)	14 (15)

Male	3 (3)	8 (8)	11 (11)

**Cell type**			

Cytotrophoblast (CTB)	4 (4)	12 (14)	16 (18)

Stromal Cells (SC)	7 (9)	16 (17)	23 (26)

Endothelial Cells (EC)	3 (5)	12 (12)	15 (17)

Hofbauer Cells (HBC)	2 (3)	14 (14)	16 (17)

Whole chorionic Villi (CV)	5 (7)	17 (17)	22 (24)

**Table 2:** Cell type-associated placental sncRNAs.

Total cell type-associated sncRNAs (*n* = 115)	Cytotrophoblast n = 10	Stromal n = 21	Endothelial n = 6	Hofbauer n = 78
miRNA (*n* = 103)	10	19	1	73
piRNA (*n* = 5)	0	2	0	3
snRNA (*n* = 4)	0	0	4	0
snoRNA (*n* = 3)	0	0	1	2
Genomic position of cell type-associated sncRNAs				
Exonic (*n* = 17)	2	4	0	11
Intronic (*n* = 39)	4	11	5	19
Intergenic (*n* = 59)	4	6	1	48

Distribution of the cell type-associated sncRNAs (*n* = 115) by sncRNA species and genomic position in all term placenta cell type samples.

## Data Availability

Raw and processed expression data and sample metadata can be accessed on GEO at accession number GSE288435. Upon a reasonable request, the corresponding authors of this article will provide unrestricted access to the original data.
